# Dementia, mood disorders, and aging: Bridging new avenues of care through shared biological pathways

**DOI:** 10.4103/agingadv.agingadv-d-26-00011

**Published:** 2026-06-18

**Authors:** Kenneth Maiese

**Affiliations:** Cellular and Molecular Signaling, New York, NY, USA

**Keywords:** Alzheimer’s disease, cell senescence, depression, diabetes mellitus, forkhead transcription factors of the “O” class (FoxOs), glucagon-like peptide-1, mechanistic target of rapamycin (mTOR), mitochondria, multiple sclerosis, oxidative stress

## Abstract

With advancing age and lifespan throughout the globe in both developed and developing nations, the risk for developing cognitive loss and mood disorders increases significantly to the extent that after reaching the age of 65, this risk almost doubles every 5 years thereafter. As a result, a corresponding rise in non-communicable diseases will impact individuals with dementia and mood disorders involving Alzheimer’s disease, multiple sclerosis, depression, and anxiety. On a clinical basis, multiple risk factors and presentations that involve the loss of intellectual capacity with the onset of mental health conditions, mood disorders preceding dementia, sleep fragmentation initiation, perivascular pathway disruptions, and circadian clock dysfunction can occur in both cognitive loss and mood disorders, but a much broader scope of shared underlying cellular pathways form the underpinning for the connection of these disorders that rests upon metabolic disorders, such as diabetes mellitus. Cognitive impairment and mood disorders can precede one another as well as coexist with related co-morbidities that involve metabolic disorders with diabetes mellitus, but present treatment strategies for these disorders are primarily symptomatic in nature and rely upon disease-altering therapies that may slow disease progression but also may be accompanied by disabling complications. Given these challenges, the institution of innovative avenues is critical at this juncture to address the mutual cellular mechanisms for the treatment of disorders involving Alzheimer’s disease, multiple sclerosis, depression, and anxiety. The pathways of cell senescence and telomere biology with aging, cellular metabolic dysfunction, apolipoprotein E, oxidative stress, programmed cell death with autophagy, ferroptosis, and pyroptosis, mechanistic target of rapamycin, glucagon-like peptide-1 receptor agonism, mammalian forkhead transcription factors of the “O” class, and mitochondrial dynamics offer a compelling potential to bridge these underlying pathways into unifying strategies for transition into efficacious clinical care for dementia and mood disorders. Tempered with this enthusiasm for these mutual disease mechanisms is the complexity of these pathways that will require meticulous oversight of the interdependence among pathway components and their ultimate biological impact on clinical outcomes.

## Introduction

In both developed and developing nations, the global population is aging at a significant rate over the past 30 years such that more than 2 billion individuals will be over the age of 60 by the year 2050. With the increased lifespan of the population, non-communicable diseases (NCDs) are rising as well, which are a result of the aging processes linked to oxidative stress, cellular senescence, and metabolic dysfunction.^1^ As a result, a significant portion of the world’s population is affected by dementia, such as with Alzheimer’s disease (AD) and multiple sclerosis (MS), and behavior and mood disorders, such as depression and anxiety. Dementia and depression are increasingly being recognized as intimately linked disorders that share underlying cellular pathways that are tied to metabolic dysfunction with disorders that include diabetes mellitus (DM). Mood disorders can surface as either clinical precursors to dementia or concurrent disorders of cognitive loss. Disorders of metabolism that are chronic in nature can become detrimental co-morbidities leading to progressive impairment of cognitive function.

Present therapies for dementia, depression, anxiety, and DM are limited in nature and most often target symptomatic care. Treatment for AD can include cholinesterase inhibitors, cannabidiol for behavioral disorders, lifestyle changes with exercise and nutrition management, wearable biosensors, and environmentally focused programs that may assist with the symptomatic care of cognitive loss, but do not halt disease progression.^2–6^ Alternatively, new immunotherapy agents approved by the United States (US) Food and Drug Administration (FDA) for the treatment of AD that can assist in the reduction of amyloid-β (Aβ) deposition in the brain offer a slower course in memory loss, but also cannot block AD progression and have the risk of brain microhemorrhages.^2^ Disease modifying therapies (DMTs) for MS offer reduction in the occurrence of relapses in relapsing–remitting MS, but cognitive and motor disabilities can continue despite a potential reduction in brain volume loss with DMTs.^7^ Similar to the treatment options for dementia, AD, and MS, therapies for depression and anxiety can include selective serotonin reuptake inhibitors, serotonin-norepinephrine reuptake inhibitors, psychotherapy, lifestyle changes with exercise and nutritional care, and structured support groups, but the overall course of disease progression can be cyclic and remain unresolved.^8–10^ Although attempting to address the overlapping clinical presentations of AD and mood disorders, treatments such as brexpiprazole that were recently approved by the FDA for the treatment of agitation in AD also remain as a symptomatic therapy.

**Figure 1 ｜:**
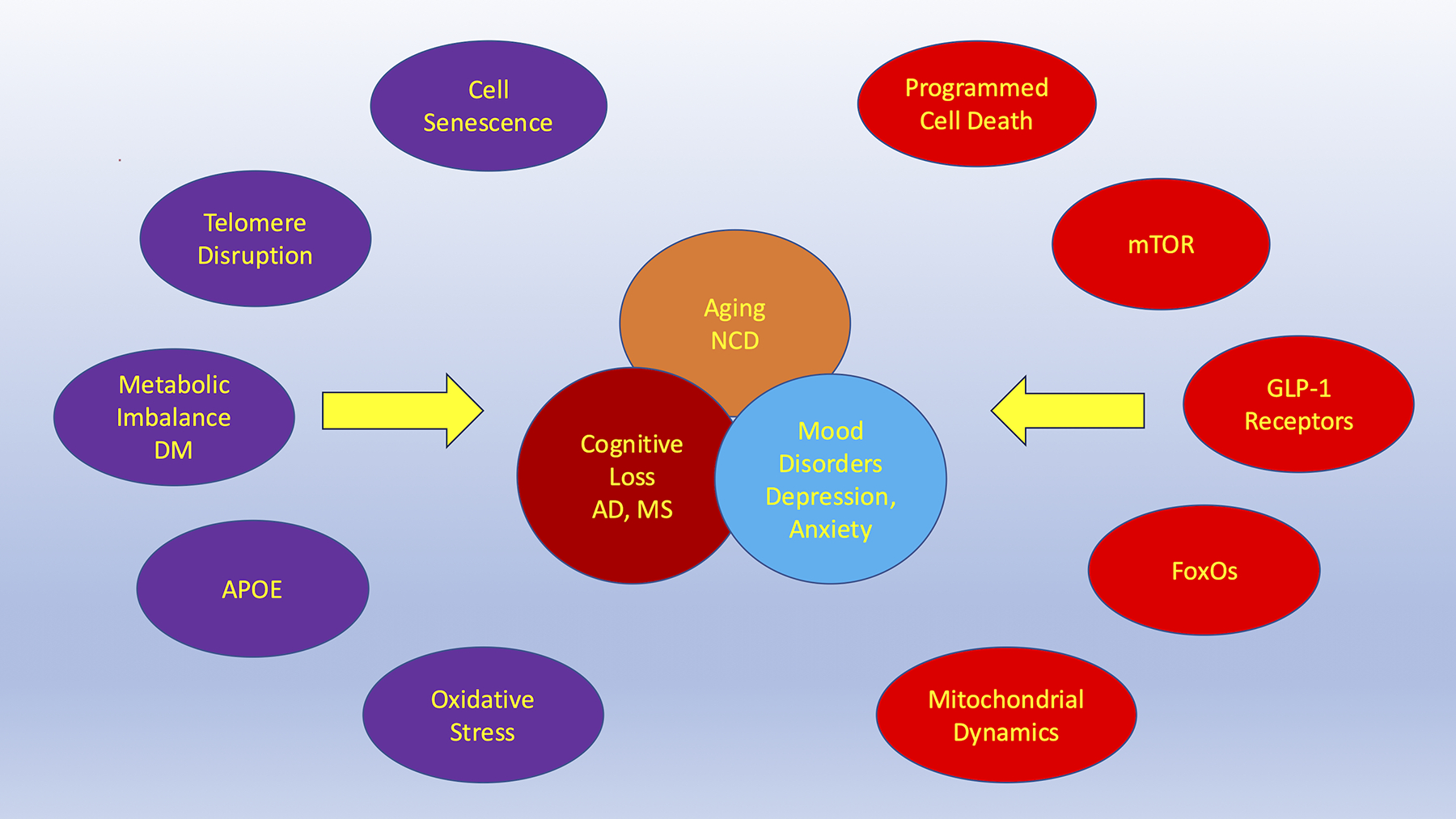
Dementia, mood disorders, and aging share critical underlying pathways that offer exciting prospects for clinical care. With the aging of the population and the increase in lifespan throughout the world, a corresponding rise in non-communicable diseases (NCDs) has resulted in the global population being impacted by dementia with Alzheimer’s disease (AD) and multiple sclerosis (MS), and behavior and mood disorders, with depression and anxiety that have led to disability, death, and financial concerns. Dementia and depression are increasingly being recognized as being intimately linked disorders sharing underlying cellular pathways that are governed through pathways of cellular metabolism that can involve diabetes mellitus (DM). The pathways of cell senescence and telomere disruption with aging, cellular metabolic imbalance with DM, apolipoprotein E (APOE), oxidative stress, programmed cell death with autophagy, ferroptosis, and pyroptosis, mechanistic target of rapamycin (mTOR), glucagon-like peptide-1 (GLP-1) receptor agonism, mammalian forkhead transcription factors of the “O” class (FoxOs), and mitochondrial dynamics offer exciting prospects to bridge these pathways into unifying strategies for clinical treatments of dementia and mood disorders.

With metabolic disorders that involve DM, attempts to achieve serum glucose homeostasis with diet care and pharmaceutical agents can modulate periods of hyperglycemia and hypoglycemia, but the course of DM will continue, and off-target effects of treatments may lead to neurovascular cell injury and organ atrophy.^11–13^ With these considerations in mind, this review focuses on avenues of inquiry that can offer innovative treatments for the aging population which involve dementia and depression offer exciting and critical treatment strategies for these disorders. Novel pathways that bridge mutual underlying disease mechanisms for these disorders and offer new insights for clinical care include cell senescence and telomere (TL) degradation mechanisms of aging, cellular metabolic dysfunction, apolipoprotein E (APOE), oxidative stress, programmed cell death with autophagy, ferroptosis, and pyroptosis, mechanistic target of rapamycin (mTOR), glucagon-like peptide-1 (GLP-1) receptor agonism, mammalian forkhead transcription factors of the “O” class (FoxOs), and mitochondrial dynamics (Figure 1).

## Data Sources

Data sources were based on a literature search using PubMed, Scopus, Web of Science, and ScienceDirect databases from January 2021 through March 2026. The search terms included “dementia”, “neurodegenerative”, “depression”, “mood disorders”, “aging”, “metabolism”, “diabetes mellitus”, “programmed cell death”, “oxidative stress”, “forkhead”, and “mitochondria” with Boolean operators (AND, OR) to focus search strategy. Peer-reviewed original research and review papers, including the citations in this review, were included while non-peer reviewed work, duplicate studies, unrelated studies, and abstracts or meeting presentations with incomplete information were excluded.

## Aging Process, Increased Longevity, Telomeres, and Cell Senescence

It is estimated that in the year 2030, approximately 15 percent of people will reach 60 years of age or be older, a significant indicator of the aging global population.^14^ By the year 2050, the number of people worldwide over the age of 60 will reach 2.1 billion, a significant increase of 1.4 billion people aged over 60 in the year 2020. In addition, individuals reaching the age of 80 or older will increase more than three times to equal 430 million individuals. As a result, the prior age limitations of 65 years in developed nations no longer apply and the expected lifespan is reaching 80 years of age for the majority of individuals.^15^ In developing countries, a rise in the number of individuals over the age of 65 will increase from 5 percent to 10 percent and by the year 2050, a majority of aged individuals will reside in low- and middle-income countries. Progressing aging of the population can be the result of several factors that lead to improvement in lifespan that include robust public policies for healthcare access and disease prevention, stronger education programs that assist with nutrition and cardiovascular health, offering improved diagnostics for acute and chronic illnesses, broad sanitation programs that can limit infectious disease, and continued identification of environmental toxins that lead to public health risks.^3,16^ In addition, other factors can alter lifespan over specific periods. For example, there have recently been decreased deaths from severe acute respiratory syndrome coronavirus 2 (SARS-CoV-2) and coronavirus disease 2019 (COVID-19) and a decline in drug overdoses that also contributed to increases in lifespan.^11,17,18^

With the onset of aging and increased lifespan, a number of conditions can develop that include cardiovascular disease, renal impairment, reproductive disorders, musculoskeletal disease, pulmonary dysfunction, and cancer.^19,20^ These conditions are related to the corresponding increase in NCDs. NCDs account for approximately 80 percent of annual deaths with over 40 million individuals dying each year and disproportionately affect lower income nations. Greater than 30 percent of individuals under the age of 60 suffer from NCDs in low- and middle-income countries, while only 10 percent of individuals in high-income countries succumb to NCDs.^21^ NCDs are chronic disorders and involve all systems of the body including disorders of cancer, pulmonary disease, trauma, cardiovascular disease, renal disorders, and suicide.^1^

**Figure 2 ｜:**
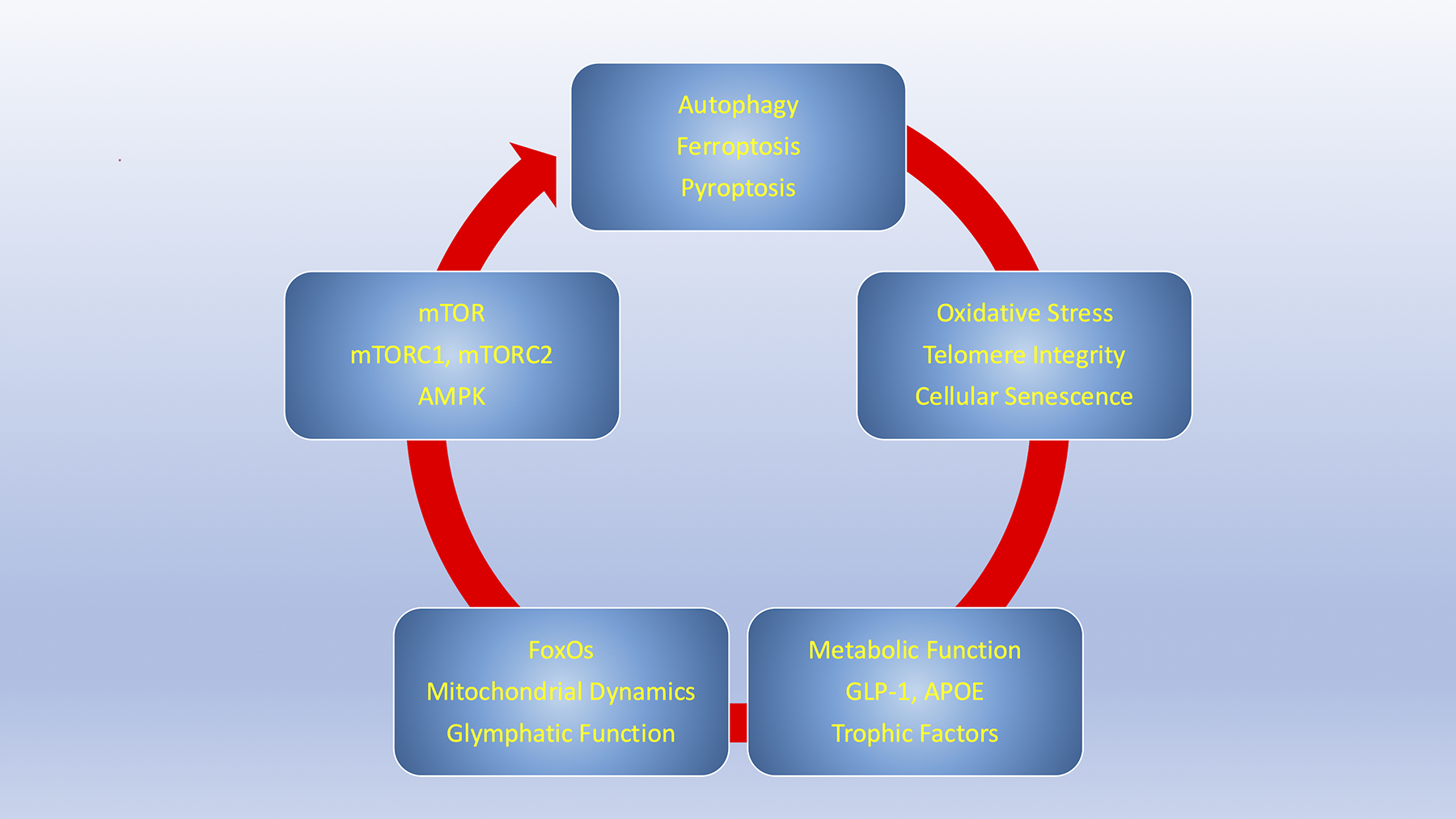
Cognitive loss, behavior disorders, and aging have common mechanistic pathways that are intimately connected to one another. Pathways of programmed cell death that involve autophagy, ferroptosis, and pyroptosis are closely tied and dependent upon multiple cellular mechanisms and cellular systems that oversee the onset and progression of cognitive loss, behavior disorders, and aging. These cellular mechanisms and systems involve oxidative stress, telomere integrity, cellular senescence, metabolic function and homeostasis, glucagon-like peptide-1 (GLP-1) receptor agonism, apolipoprotein E (APOE), trophic factor oversight of autophagy, the mechanistic target of rapamycin (mTOR), mammalian forkhead transcription factors of the “O” class (FoxOs), mitochondrial dynamics with mitophagy and mitoptosis, glymphatic function of the brain, and the components of the mTOR pathway with mTOR complex 1 (mTORC1), mTOR complex 2 (mTORC2), and AMP activated protein kinase (AMPK).

Cellular mechanisms that are affected by aging and lead to chronic disorders involve TLs (Figure 1). These complexes of DNA are present at chromosome ends and oversee protection for genomic DNA, cellular replication, and cellular survival. TLs consist of more than 2000 repetitions of non-coding double-stranded DNA with the sequence “TTAGGG” and are completed with a guanine rich single-stranded DNA. The protein complexes telosome, shelterin, and CTC1-STN1-TEN1 (CST) oversee TL activity and stability.^22^ Telomerase protein is activated during cell division to add tandem repeat ribonucleic acid (RNA) templates for TL length maintenance, since approximately 25–200 base pairs are normally lost in this process. Despite these protective mechanisms, telomerase function can lose viability or TLs can become non-functional once they have less than 500 base pairs. At this point, cellular replication fails and cellular senescence ensues (Figure 2).^5,23^

With the onset of cellular senescence, aging processes can develop into chronic inflammation, cellular injury in multiple systems progress to involve neurons, microglia, endothelial cells, cardiomyocytes, fibroblast, and pathways leading to fibrosis and programmed cell death.^24^ Subsequently cellular energy homeostasis is lost, the generation of reactive oxygen species (ROS) ensues with oxidative stress, and mitochondrial dysfunction results.^25^ In fact, ROS generation also occurs with TL shortening and the development of cellular senescence.^19,23^ These pathways of cellular senescence become critical mediators of the NCDs involving cognitive loss and dementia, behavioral disorders with depression, and metabolic disorders (Box 1).

## Dementia, Mood Disorders, and Metabolic Dysfunction

A large proportion of the global population is impacted by cognitive loss that is linked to nervous system disorders such as AD, behavior and mood disorders such as depression, and metabolic disorders, such as DM. There exist over 600 nervous system disorders that affect 3 billion individuals worldwide, a 42 percent representation of the global population, and yield 35 million disability-adjusted life years.^21,26^ Although dementia is underdiagnosed and has delays in care that can exceed more than 18 months, dementia is now the 7^th^ leading cause of death per the World Health Organization.^26^ Costs for dementia care increase by 42 percent annually with over 2 trillion United States dollars (USD) spent in the year 2019.^5,26^ As the population ages, significantly increased social services as well as medical care will be required for the care of individuals with dementia. It is predicted for the need to employ over an additional 70 million social care individuals and healthcare members for the expected 160 million individuals globally that will develop dementia and AD by the year 2050.^26^

In regard to AD, this disorder represents at least 60 percent of the individuals with dementia who have the sporadic form of AD, are over 65 years of age, and equals almost 11 percent of the population. Currently, at least 7 million individuals in the US have AD and this is expected to increase to 30 million individuals by 2050.^7,27^ In contrast to the sporadic form of AD, familial AD usually occurs prior to the age of 55 and represents a mutation of amyloid precursor protein (APP) gene. Familial AD has variable single-gene mutations on chromosomes 1, 14, and 21 and affects approximately 200 families globally. In addition to AD, other prevalent neurological disorders are now being increasingly recognized to lead to cognitive loss in patients. For example, MS, a demyelinating disorder that affects almost 3 million individuals globally, can lead to cognitive loss. Women are usually affected by MS more often than men, but loss of cognition with MS occurs in both genders and can be progressive with word finding difficulties and impairment in object naming.^7,28^ Almost 65 percent of individuals with MS have difficulty with executive functions that involve attention, information recall, and memory. Cognitive impairments in MS exist with word finding difficulty and visual object naming.^29^ The reduction in cortical processing speed with cognitive loss in MS may be a consequence or independent of mood disorders.^29^ Similar to AD, the cognitive impairments and mood disorders in MS patients can prevent individuals from returning to previous functioning and the workforce.^30^ In cerebrospinal fluid examinations, changes in A42 as seen in AD also may signal early cognitive decline in MS.^31^ Tau, which leads to AD pathology, also occurs in individuals with MS^32^ and tau can lead to oligodendrocyte maturation impairment to result in demyelination.^33^ The onset of dementia in neurodegenerative disease can also be a result of the TL shortening and cell senescence (Figure 1). Some individuals with leukocyte TLs shortening can experience an increased risk of dementia and AD.^34^ The converse has also been noted. Longer length of TLs on cerebral magnetic resonance imaging has been suggested to reduce dementia onset since increased grey matter volumes in the hippocampus, lower volume of white matter hyperintensities, and decreased basal ganglia iron are present on magnetic resonance imaging with preserved TLs.^35^

Behavioral mood disturbances that involve depression and anxiety are increasingly being recognized as either symptomatic precursors or concurrent disorders with nervous system disorders involving dementia, such as with AD and MS. Anxiety can trigger inflammation in the nervous system that results in memory loss and ultimately long-term disability with AD or MS. Recent studies further support this premise of the link between cognitive impairment and depression such that individuals with intellectual and developmental disabilities suffer from a higher rate of mental health conditions.^8^ Depression can also be associated with the early onset of neurodegenerative disease such that during the initial stages of MS, 30 percent of patients may suffer from depression that is associated with increased demyelinating disease.^36^ Depression and disorders that include anxiety affect more than 7 percent of the global population.^15^ Although during pandemics, the number of individuals impacted by depressive behavior can increase, at least 330 million individuals can suffer from depression and anxiety.^37^ Depression is almost 50 percent more frequent in women than men and can result in reduced activity, despair, and hopelessness while anxiety results in distress, tension, and fear of potential future threats. Both depression and anxiety can lead to rapid or irregular heartbeat, restlessness, gastrointestinal distress, nausea, fatigue and cognitive loss. With advancing age, the risk for depression increases over the age of 65, but of note, suicide is the 4^th^ highest cause of death in the ages of 15 to 29 years old.^38^

A number of factors may tie dementia and cognitive loss to the behavioral disorders of depression and anxiety. Sleep disturbances and sleep fragmentation that accompany depression and anxiety are a potential risk factor for the onset of cognitive loss such as in AD and MS. An important component of the nervous system that can be affected by sleep disturbances is the perivascular pathway (Figure 2).^9,11^ The perivascular pathway oversees the flow of cerebrospinal fluid that showers the brain through a perivascular network and eliminates toxins to include Aβ, tau, and α-synuclein. During sleep impairment with sleep fragmentation or sleep deprivation, dysfunction in the circadian rhythm results with failure to remove toxins from the brain leading to cognitive loss. Sleep deprivation can result in neuroinflammation, cardiovascular disturbances, gut dysbiosis, programmed cell death, and impairments in circadian rhythm regulation.^11,39^ The loss of circadian rhythm has been associated with the development of AD, depression, and metabolic disorders.^9,12,21,40^ Sleep disorders with loss of proper circadian rhythm also may affect cognition through increased risk for COVID-19 infection that can develop into long-COVID, also known as long-haul COVID, chronic COVID-19, or post-acute COVID.^17,41,42^

Dementia and depression are also intimately linked through metabolic pathways that include DM (Figure 1). As the population ages with extended lifespan, metabolic disorders are increasing in prevalence as well. As a result, mortality for DM has increased by a large extent as well to over 20 percent during the prior 25 years when compared to other NCDs that involve cancer, respiratory disorders, and cardiovascular disease.^15^ Approximately 2 million deaths annually are attributed to DM, with 50 percent of the deaths occurring in individuals prior to age 70. As of the year 2022, greater than 800 million worldwide individuals are estimated to have DM.^15^ At least 14 percent of individuals over 18 years of age suffer from DM, an increase from 7 percent in 1990. Interestingly, an additional significant number of individuals are believed to suffer from metabolic disease or at least have an increased risk for the development of metabolic disorders, but remain without a diagnosis.^15^ In individuals over the age of 18 years, 10 million may not be correctly diagnosed as having DM and greater than 40 percent of individuals in the US may have prediabetes due to elevations in their fasting glucose and hemoglobin A1c parameters.^11^

Metabolic disorders that include DM are chronic and progress to affect all organs of the body. Metabolic disease can result in renal impairment, hepatic disease, cardiovascular disorders, retinal disease, central and peripheral nervous system disease, musculoskeletal disease, and tumorigenesis.^43,44^ Given the impact of disorders such as DM on the entire body, the financial implications for the care and treatment of DM also present a number of challenges. Costs for DM care in 2022 were over 400 billion USD and annual costs for patients with complications can exceed 22 thousand USD per year. If one also considers costs for disability and functional care loss in patients with DM, financial considerations exceed 760 billion USD and are greater than 2 percent of the Gross Domestic Product.^15^

Factors that lead to risk onset and progression of DM are multifactorial in origin and can include lower education level, socioeconomic status, high serum cholesterol, hypertension, consumption of alcohol and tobacco, infection with SARSCoV-2, lack of physical activity, and obesity.^11,12,42^ With these risk factors, education level appears to be important and may be a factor for overall care and medication compliance with 13 percent of DM individuals having less than a high school education while less than 7 percent of DM individuals are affected who have greater than a high school education level.^19^ Obesity also affects multiple parameters for DM that involve glucose intolerance, oxidative stress, insulin sensitivity, cerebral blood flow, inflammation, stem cell function, longevity and aging, mitochondrial function, and infection susceptibility, such as with COVID-19.^11,45^

In regard to cognitive loss and depression, metabolic disorders, such as DM, can become serious co-morbidities that lead to nervous system injuries (Box 1). In the peripheral nervous system with DM, more than 75 percent of individuals can be compromised by autonomic dysfunction or peripheral neuropathies that can be detected by elevated neurofilament light chain (NFL) serum levels.^11,46^ Autonomic dysfunction as a risk factor can lead to cognitive impairment in AD, Parkinson’s disease, and vascular dementia. Patients with diabetic retinopathy also have an increased risk for dementia that includes both AD and vascular dementia.^47^ DM promotes Aβ and tau deposition in the brain that can lead to AD onset, progression, and memory loss.^11,48^ During DM, circadian rhythm dysfunction with sleep fragmentation can result in memory impairment associated with AD that also affects the course of DM.^12,21^ Insulin resistance can result in susceptibility to Aβ and tau toxicity, leading to inflammatory pathways that can affect protein kinase activity and neuronal signaling, and promote dysfunction in programmed cell death pathways that involve autophagy.^11,49^ Elevations in FoxOs and high mobility group A1 (HMGA1), a chromatin-binding protein, may promote memory loss in AD during DM 4 (Figure 2). In DM, vascular cells of the brain are at risk, which can result in endothelial dysfunction and memory loss.^11,50^ Furthermore, obesity tied to metabolic disease can alter the function of endothelial cells and the permeability of the blood-brain barrier, which can lead to vascular dementia.^51^ Diffuse cortical dysfunction can occur over the long-term with DM, as evidenced by raised serum NFL levels that indicate neuronal cell loss. Thus, NFL may serve as an early diagnostic parameter for disease progression.^46,52^

As a chronic disorder, DM can alter neuroplasticity of the brain, potentially leading to depression. DM has been shown to reduce growth factor expression, such as insulin-like growth factor-1, that can lead to prefrontal cortex damage in experimental models and contribute to anxiety.^53^ The loss of growth factor expression in the brain, such as with erythropoietin (EPO) and in the presence of DM, can foster programmed cell death with apoptosis, autophagy, inflammation, and depression.^41^ During DM, EPO can have an anxiolytic effect, limit inflammatory cell responses, improve verbal memory and potentially improve mood, as well as alter pathways through protein kinase B (Akt B) and mTOR to limit depression and mood alterations. Trophic factors, such as EPO, rely on pathways of Akt and mTOR to foster neuronal and vascular cell survival to maintain cognitive function.^54,55^ EPO can reduce social stress in animal models and requires mTOR activity to limit depression. mTOR, a 289-kDa serine/ threonine protein kinase that is also known as the mammalian target of rapamycin and the FK506-binding protein 12-rapamycin complex-associated protein 1, is a central component for depression regulation and is formed from mTOR Complex 1 and mTOR Complex 2.^11,23^ Activation of mTOR enables antidepressants to promote decreased depression and anxiety.^56^ mTOR modulates cellular metabolism through the AMP activated protein kinase (AMPK) during DM that may be an important pathway for the onset of clinical depression and anxiety (Figure 2).^57^ In conjunction with the growth factor brain-derived neurotrophic factor, mTOR may modulate the onset of depression in women through estrogen pathways^58^ and loss of mTOR may be a factor in the promotion of anxiety and affect hyperexcitability of the amygdala.^59^

## Apolipoprotein E, Oxidative Stress, and Autophagy in Dementia and Depression

Several underlying cellular metabolic pathways are intimately tied to the development of neurodegenerative disorders that lead to dementia and depression (Box 1). The ε4 allele of APOE (APOE-ε4) gene is one such pathway.^7,11^ APOE is generated by hepatic cells and is required for cellular metabolism to modulate lipid homeostasis involving cell transport of cholesterol, phospholipids, and triglycerides.^41,42^ APOE in the central nervous system begins its production in astrocytes and oversees cholesterol transfer to neurons through APOE receptors. In individuals who harbor two *APOE-ε4* alleles and are homozygous ε4/ε4, the risk of developing AD dementia by age 85 is increased by more than twenty times. *APOE-ε4* also may be associated with cognitive loss in patients with MS. *APOE-ε4* in patients with MS demonstrate delays in responding to stimuli^60^ and those with optic neuritis that is present in 50% of patients with MS have elevated APOE serum levels.^61^ In addition, the *APOE-ε3/ε3* genotype may increase the risk in males for the development of optic neuritis.^62^ Through APOE, Aβ deposition in the brain can be reduced through programmed cell death pathways of apoptosis that involve phosphatidylserine membrane exposure.^11,40^ Subtypes of APOE that are not part of *APOE-ε4*, such as *APOE-ε2* alleles, can prevent Aβ aggregation during phosphatidylserine membrane exposure. However, APOE-ε4 does not block A aggregation and deposition of Aβ occurs leading to cognitive loss and AD.^41,63^ In addition, *APOE-ε4* has other risks for cognitive loss with AD and MS (Figure 2). In optic neuritis which occurs in almost 50 percent of patients with MS,^61,64^ APOE serum levels can be elevated and the *APOE-ε3/ε3* genotype can increase the risk for optic neuritis development in males.^62^ *APOE-ε4* may facilitate viral antigen infection and lead to cerebral microhemorrhages during SARS-CoV-2 exposure with COVID-19 that results in cognitive loss and potential complications with AD.^11,42^ As a mechanism mediated by chronic metabolic dysfunction, progressive cognitive loss can occur during long-COVID, also termed long-haul COVID, chronic COVID-19, or post-acute COVID.^17,41^ In relation to MS, SARS-CoV-2 infection may lead to higher death rates in MS patients.^7,65^ Cognitive impairment with COVID-19 and *APOE-ε4* also has been associated with higher rates of depression.^42^

Loss of cellular metabolic homeostasis fosters the generation of ROS and oxidative stress during dementia and depression (Figure 1).^9,41,66^ Under conditions that maintain cellular function, the presence of ROS is tempered by several antioxidant systems. The vitamins K, B, E, C, and D, superoxide dismutase, catalase, and glutathione peroxidase function within cells to limit oxidative stress generated through ROS by the free radical species of singlet oxygen, nitric oxide, superoxide free radicals, hydrogen peroxide, and peroxynitrite. Once antioxidant systems become ineffective to limit the effects of ROS, such as during metabolic disorders, processes of cellular senescence, TL dysfunction, circadian clock gene impairment, and stem cell loss ensue.^22,45,67^ As a result, cell injury occurs in neuronal cells, vascular endothelial cells, microglia, astrocytes, and myelin-producing oligodendrocytes that can affect synaptic plasticity, growth factor loss, and neurotransmitter function to promote the onset and progression of dementia with AD and MS, as well as depression and anxiety.^7,9^

Metabolic disease, such as DM, and oxidative stress can both lead to the induction of programmed cell death with autophagy (Figure 2).^67,68^ Autophagy, which usually involves macroautophagy that recycles organelles through autophagosomes, can offer cellular protection.^40,43^ Through pathways that involve induction of autophagy, cell survival and mitochondrial integrity can be preserved with reductions in oxidative stress and apoptotic cell death. Induction of autophagy oversees β-cell proliferation in the pancreas^69^ and limits insulin resistance with inflammatory high serum lipids in obesity models of autophagy *Atg7* gene deletion.^70^ Alterations in DM management with a focus on nutrition, diet, flavonoids, fatty acids, obesity, gut microbiota, and exercise can result in activation of autophagy and the maintenance of cellular metabolic homeostasis.^10,14,21^ In particular, exercise may also influence mitochondrial dynamics and mitophagic flux to offer increased cellular survival.^14,71^ However, a balance in the activity of autophagy during metabolic disease is necessary since under some conditions of hyperglycemia, autophagy dysregulation occurs, which leads to cell injury.^22,72^ Hyperactive autophagy pathways can affect stem cell survival and mitochondrial function, which further promotes oxidative stress.^11,73^ In addition, heightened activity of autophagy can foster diabetic retinopathy,^74^ block cerebral interneuron progenitor cell growth,^75^ prevent neuronal cell repair,^76^ and worsen cognitive function.^67^ Pathways that can modulate autophagy activation, such as mTOR, are essential under such conditions to preserve cellular survival. As evidence, protection by trophic factors that include EPO employs the activation of mTOR pathways and at times limits autophagy induction to protect neurons and microglia from oxidative stress.^11,54,77^

During activation of autophagy, memory retrieval and behavior can be improved during dementia and depression (Box 2). Loss of autophagy in dementia models and DM leads to progressive loss of memory. Activation of autophagy in experimental models can reduce oxidative stress, increase survival of neurons, limit A deposition in the brain, preserve mitochondrial function, and prevent cognitive impairment.^41,78,79^ On the converse side, autophagy inhibition can result in brain tau and A accumulation.^11,80^ In MS, autophagy can modulate oligodendrocyte development, microglia activation, and myelination,^7,81^ In depression and anxiety, experimental models of major depressive disorder demonstrate significantly elevated transcription of autophagy-related genes Atg6, Atg7, and Atg12 in the prefrontal cortex under repeated social defeat and that enhanced autophagic flux in the prefrontal cortical microglia reduced depression.^82^ Anti-depressive treatments, such as with melatonin, rely on the presence of autophagy since depression resumes during autophagy inhibition.^38^ In models of anxiety, induction of autophagy with reduction of oxidative stress was associated with improved reduction in behavioral abnormalities.^83^ Disorders with depressive and anxiolytic symptoms, such as autism spectrum disorder, also may require induction of autophagy pathways to overcome dysregulated mTOR signaling in both excitatory and inhibitory neurons.^66^

Yet, without fine controls over autophagy flux levels, the beneficial effects of autophagy induction can be lost (Figure 1). Autophagy may play a negative role in MS during exposure to infectious agents, such as SARS-CoV-2, since COVID-19 can result in increased death rates during MS.^7,65^ Autophagy induction may lead to viral host susceptibility through the activation of lysosome-mediated suppression of intrinsic immunity. With elevated levels of autophagy, tauopathy also can progress and lead to increased neuronal damage (Box 2).^80^ Exaggerated autophagy levels also have been associated with increased depression that requires modulation through mTOR pathways to reduce depressive-type phenotypes.^10^ In the presence of advanced glycation end products (AGEs) with DM and autophagy activation, endoplasmic reticulum stress can occur^84^ with the promotion of oxidative stress.^11,85^ In conjunction with hyperactivity of autophagy, pathways of ferroptosis and pyroptosis also may influence clinical outcomes. Ferroptosis occurs with the loss of glutathione homeostasis as a result of cellular iron accumulation^19,86,87^ and leads hippocampal neuronal injury during epilepsy,^86^ Parkinson’s disease,^87^ and dementia.^11,88^ Pyroptosis oversees inflammasomes, and activation of caspase 4, caspase 5, and caspase 1 to lead to cortical neuronal death and dementia through tau pathology and microglial inflammation (Figure 2).^11,87^

## Glucagon-Like Peptide-1 Agonism, Forkhead Transcription Factors of the “O” Class (FoxOs), and Mitochondrial Dynamics in Neurodegenerative Disease and Mood Disorders

Given the complex role of programmed cell death that involves autophagy and related pathways of ferroptosis and pyroptosis with the metabolic basis of neurodegenerative disease, it is intriguing to consider how recently approved US FDA DM treatments for glucose homeostasis and obesity intersect with the onset of dementia and depression. Of interest are GLP-1 receptor agonists, such as semaglutide, tirzepatide, and liraglutide, which may offer broader applications beyond serum glucose management for the care of dementia, AD, MS, depression, and anxiety.^5,10^ Currently, metabolic disorders exclusive of DM involving nonalcoholic fatty liver disease, also known as metabolic dysfunction-associated steatotic liver disease, are FDA approved for treatment with GLP-1 agonists.^5^ GLP-1 receptors are G protein-coupled receptors that involve proteins across membranes (Figure 1). These proteins include 7 α-helical transmembrane domains, an extracellular N-terminus, and an intracellular C-terminus and are present on several cell types that include nerve cells, cardiac cells, chondrocytes, hepatic cells, and pancreatic β-cells. GLP-1 agonists are active in the nervous system and have been shown to reduce retinopathy during DM.^89^ Stimulation of GLP-1 receptors can be mediated by the transient receptor potential cation channel subfamily V member 2 channel activation and the transient receptor potential cation channel subfamily V member 1 family receptors are effective mediators to modulate cellular metabolism, oxidative stress, inflammation, and cellular survival.^7,90^ Agonism of GLP-1 receptors can reduce hyperglycemia^91^ and protect cells from DM oxidative stress.^92^ In degenerative disorders of the brain that can lead to memory loss, GLP-1 receptor agonism may prevent neuronal cell demise as monitored with reductions in brain immunostaining of NFL.^93^ In experimental models of DM encephalopathy, activation of GLP-1 receptors is protective against neuronal cell injury that is mediated in this case through Akt, mTOR, and the modulation of autophagy induction.^92,94^ During environments of cholesterol toxicity, GLP-1 activation blocks cholesterol-induced apoptotic cell death through mTOR activation (Figure 2).^5,95^

The protection of neurons with GLP-1 agonism extends to experimental models that involve improvement of cognitive and motor function.^94^ GLP-1 activation also results in protection of vascular cells, reduction in oxidative stress, and maintenance of mitochondrial function.^5,13^ Similar to the potential beneficial effects of GLP-1 receptor agonism with maintaining cognition and limiting dementia, experimental models of depression with associated cognitive loss support the role of GLP-1 receptor activation to reduce depression and improve cognition through pathways that activate Akt, mTOR, and block heightened autophagy induction.^10^ Yet, cellular protection that can be offered by GLP-1 agonism does not consistently function through mTOR activation but employs autophagy induction with a corresponding reduction in mTOR activity (Box 2). Treatment of DM retinopathy with GLP-1 agonism requires autophagy induction.^89^ As a result, it is clear that pathways involving GLP-1 agonism in relation to the neurodegenerative disorders of dementia and depression require a clear focus on the fine modulation of autophagy flux levels to achieve desired clinical outcomes. These observations also may provide insight to some other limitations of GLP-1 agonist treatments that involve gastrointestinal symptoms such as diarrhea, nausea, and vomiting, nonarteritic anterior ischemic optic neuropathy, alopecia, acute pancreatitis, and depression.^96,97^

Mammalian forkhead transcription factors of the “O” class (FoxOs) play a critical role in cell senescence, cell death, metabolism, oxidative stress, and aging especially in neurodegenerative and mood disorders (Figure 2).^11,98^ In the forkhead family, greater than one hundred genes and nineteen human subgroups have been described. These genes are *FOXA* to *FOXS* after the discovery of the *Drosophila melanogaster gene forkhead*. Forkhead proteins are also known as forkhead in rhabdomyosarcoma (FKHR) (FOXO1), FKHRL1 (forkhead in rhabdomyosarcoma like protein 1) (FOXO3a), the *Drosophila* gene fork head (*fkh*), Forkhead RElated ACtivator (FREAC)-1 and −2, and the acute leukemia fusion gene located in chromosome X (*AFX*) (*FOXO4*). Mammalian FOXO proteins of the “O” class have the members that include FOXO1, FOXO3, FOXO4, and FOXO6. FoxO proteins and their functions are conserved among several species that include *Caenorhabditis elegans, Drosophila melanogaster*, and mammals. In *Caenorhabditis elegans*, FoxO proteins are homologous to the transcription factor DAuer Formation-16 (DAF-16) which can modulate insulin signaling, cell cycle regulation, cell survival, and extension of lifespan.^7^ FoxO proteins, as transcription factors, bind to DNA through the FoxO-recognized element in the C-terminal basic region of the forkhead DNA binding domain with 14 protein-DNA contacts overseeing gene expression of targets in the -helix H3 recognition region. Control of the interaction between FoxOs and DNA can involve protein phosphorylation, protein acetylation, FoxO nuclear compartmentalization, and changes in electrostatic changes.^7,99^ Akt can phosphorylate FoxO proteins, lead to mTOR activation, and ultimately block FoxO nuclear translocation through cytoplasmic association with 14-3-3 proteins to prevent caspase activation and apoptotic cell death.^67,100^ Under some scenarios, Akt, in conjunction with agents that involve nicotinamide, can also maintain FoxO3a protein integrity to block FoxO3a proteolysis and prevent the subsequent generation of “pro-apoptotic” amino-terminal (Nt) fragments that lead to cell death.^19,101–103^

FoxOs oversee multiple cellular pathways that involve aging, dementia, and depression (Figure 1). In models of advanced maternal aging that can contribute to adverse pregnancy outcomes, FoxO1 activity leads to cell senescence and impaired placental development.^104^ Age-related neuronal cell loss that results from cell senescence has been linked to loss of Akt activation with increased FoxO1 function.^105^ Vascular endothelial cell senescence that impairs endothelial function during DM is overseen by FoxO1 activity and can be reversed with agents that enhance autophagy pathways, such as metformin treatment.^106^ Interesting, metformin also improves cognitive function in models of DM through the inhibition of the FoxO6 pathway.^98^ In neuronal exposure to oxidative stress and inflammatory toxins, cell injury is reduced with the blockade of FoxO3 activity.^107^ Trophic factors, such as EPO, also provide cellular protection with the oversight of FoxO3a, FoxO1, and autophagy activity (Figure 2).^21,54,108^ EPO promotes the cytoplasmic sequestration of FoxO with 14-3-3 protein to block forkhead transcription and increase cellular survival during oxidative stress. Prevention of hippocampal FoxO1 activation in models of DM can improve memory function, 99 removal of FoxO in models of AD can limit toxicity from Aβ exposure,^109^ experimental models of major depression are resolved with control of FoxO3a dysregulation,^110^ nerve injury and cognitive impairment is lessened with inactivation of FoxO1,^76^ and inflammatory pathways with microglia are tempered with inhibition of FoxO pathways.^7,111^

Careful modulation of cellular FoxO pathways can be critical to overall cell survival in the nervous system, since FoxOs can also be supportive and necessary to foster cellular growth and maintenance (Box 2). FoxO activation may be required to regulate innate immunity against infectious agents^112^ while loss of FoxO and absence of autophagy induction can lead to disorders of development.^7,113,114^ In addition, neuronal Huntingtin (mHtt) protein deposition is reduced with increased activation of FoxO1 in experimental studies with Huntington’s disease,^115^ atherosclerosis is limited with activation of FoxO1 and autophagy,^116^ cell survival is increased during activation of FoxO3 and the control of ferroptosis,^19,117^ myelination in the brain and oligodendrocyte growth that can limit MS is dependent upon FoxO1,^118^ and tumor cell growth can be controlled through FoxO3 upregulation.^119^

The maintenance of mitochondrial integrity and function becomes significant in the intricate association with cellular metabolism, programmed cell death FoxOs, and the onset and progression of cognitive loss and depression (Figure 1). As cellular organelles that are under 3 μm^2^ in cross-sectional diameter with a double membrane composition, mitochondria are present in plant, fungi, animals, and cells that require aerobic respiration to generate adenosine triphosphate (ATP).^11,79^ Although mature red cells lack mitochondria, remaining cells in the body may harbor thousands of mitochondria that are involved in clock genes and circadian rhythm, cellular metabolism, oxidative stress, and programmed cell death with autophagy.

With programmed cell death involving mitochondria, the processes of mitophagy and mitoptosis, programmed fragmentation of mitochondria through caspase-mediated pathways, can occur that remove damaged mitochondria to offer cellular function protection during oxidative stress (Box 1). Mitophagy and the removal of damaged or unnecessary mitochondria can also occur during elimination of parental mitochondria, cell differentiation, and to promote cellular energy homeostasis that could be protective against neurodegenerative disorders and mood disorders.^41,120^ Activation of mitochondrial autophagy pathways with mitophagy can, in experimental models, reduce sleep fragmentation that has the potential to improve cognition, block oxidative stress, and limit apoptotic neuronal cell death through mTOR signaling with AMPK (Figure 2).^78^ During periods of metabolic dysfunction and lipotoxicity, mitophagy also may be beneficial to activate necessary pathways of autophagy that can include FoxOs to maintain glucose homeostasis.^121^ Furthermore, FoxO3a can modulate energy stress signaling through mitochondrial activity during loss of cellular metabolic homeostasis.^57^ In regard to cognitive dysfunction, such as with AD, loss of mitochondrial activity is associated with increased Aβ toxicity in the brain, mitochondrial membrane depolarization, FoxO activation, and programmed cell death of neurons, astrocytes, and microglia.^7,122–124^ Preservation of cognitive function through mitochondrial activation appears to be dependent, at least in part, on GLP-1 receptor activation^5,13,20,93^ as well as on the presence of APOE for the maintenance of neuronal synaptic function.^11,125^ Experimental models of depression, anhedonia, and cognitive loss in MS also illustrate the need to modulate energy stress, control autophagy flux with the activation of FoxOs, and maintain mitochondrial activity to reduce glutamate excitotoxicity, oxidative stress, and programmed cell death such as with ferroptosis.^7,126^ Studies also have identified mitochondrial and programmed cell death-related genes in mood disorders, such as obsessive-compulsive disorder, for early diagnosis and potential identification of future treatments.^127^

## Limitations

The studies and investigations presented are considered novel and emerging for developing new clinical perspectives for the treatments of dementia and depression that includes disorders of AD, MS, depression, and anxiety, and therefore a clear understanding of some of the present limitations and challenges of this body of work should be noted. First, the underlying metabolic pathways that link dementia and depression together are complex and require further understanding. Homozygous *APOE-ε4* alleles can significantly increase the risk for developing AD, may lead to cognitive deficits in patients with MS, and may facilitate viral antigen infection that is associated with cognitive loss, but other types of APOE, such as those with *APOE-ε2* alleles, can offer cellular protection through the prevention of Aβ aggregation, illustrating our knowledge of these pathways require further development. Second, programmed cell death pathways with autophagy, ferroptosis, and pyroptosis are complex, and require further elucidation how these pathways can be modulated for effective clinical care. Induction of autophagy can limit oxidative stress, improve neuronal survival, depress Aβ deposition, maintain mitochondrial function, and assist with oligodendrocyte function and axonal myelination to block cognitive impairment and limit depression and anxiety. Yet, heightened levels of autophagy promote viral antigen infection, foster oxidative stress and endoplasmic reticulum stress, lead to mitochondrial dysfunction, and result in cognitive loss and depression. Pathways, such as the mTOR pathway, become critical for regulating autophagy and can be mediated by trophic factors, such as EPO and GLP-1 receptor agonists (Box 2). Additional work needs to advance to gain greater understanding of these intimately connected pathways that can require varying levels of autophagy or mTOR activity. These pathways also may be cellular and disease specific since even applications of GLP-1 agonism can require mTOR inhibition with autophagy activation when directed toward disorders such as DM retinopathy. Third, given the novel foundation of these pathways for clinical care, future work will need to translate the current knowledge of *in vitro* and *in vivo* animal models to clinical applications in well-defined patient based trials to further these underlying pathways for dementia and mood disorders into effective and safe clinical care. Current clinical studies, even those that suggest cortical loss during metabolic dysfunction with DM through the monitoring with new diagnostics such as NFL will require broader patients studies that take into account patient co-morbidities that can affect clinical observations in different sub-populations of patients. These limitations can serve to highlight the challenges ahead to further clinical translation of novel pathways for dementia and mood disorders into patient care.

## Conclusion

As a result of multiple factors that include robust public health policies, improved nutrition and sanitation, and development of sophisticated diagnostics for early disease detection, lifespan with increased age has progressed remarkably, with expectations that greater than 400 million individuals will reach the age of 80 or older within another two decades. Yet, with the aging of the population and this rise in lifespan is an increase in NCDs that account for over 80 percent of the annual deaths with a significant portion of the population impacted by dementia, such as in diseases with AD and MS, and mood disorders, such as with depression and anxiety. Interestingly, these disorders share mutual clinical attributes and underlying cellular pathways that are linked through pathways of cellular metabolism that can involve DM. Loss of cognition and memory in individuals is frequently accompanied by mood disturbances of depression and anxiety. Yet, present therapies for neurodegenerative cognitive loss, mood disorders, and metabolic dysfunction are directed primarily toward symptomatic care, given that these diseases will continue to progress regardless of care directives. As a result, innovative therapeutic strategies that examine mutual underlying cellular pathways of these disorders are highly warranted to fill this gap in clinical care. The investigation of the pathways of cell senescence and TL degradation with aging, cellular metabolic dysfunction, APOE, oxidative stress, cell death pathways of autophagy, ferroptosis, and pyroptosis, mTOR, GLP-1 receptor agonism, FoxOs, and the dynamics of mitochondrial integrity and function offer exciting prospects for the development of therapeutics that can address not only disease onset, but also disease progression with AD, MS, depression, and anxiety. These pathways are complex and highly interconnected, such that an in-depth understanding of the modulation of individual pathway components will be required for the fruitful translation to clinical medicine.

## Box 1: Article Highlights

- Aging of the global population has progressed rapidly with the expectation that over 2 billion individuals will be over the age of 60 in the next 20 years with improved healthcare access, strong nutritional and cardiovascular health programs, and sophisticated disease diagnostic programs
- With advanced aging, lifespan extension has led to a corresponding rise in non-communicable diseases (NCDs) that impact the global population with dementia through Alzheimer's disease (AD) and multiple sclerosis (MS) and mood disorders with depression and anxiety.
- Dementia and mood disorders share clinical risk factors with intellectual disability tied to mental health development, sleep fragmentation onset, glymphatic pathway disruptions, and circadian clock dysfunction, but the foundation for the shared underlying cellular pathways in these diseases rests upon metabolic disorders that can involve diabetes mellitus (DM).
- Although dementia, mood disorders, and DM are believed to be under diagnosed, have significant implications for disability, death, and financial burden, and have multiple risk factors that can be associated with education level, socioeconomic status, and lack of physical activity, current therapeutic care plans are symptomatic in focus and do not halt disease progression.
- Targeting novel pathways of cell senescence and telomere (TL) biology with aging, cellular metabolic dysfunction, apolipoprotein E (APOE), oxidative stress, programmed cell death with autophagy, ferroptosis, and pyroptosis, mammalian target of rapamycin (mTOR), glucagon-like peptide-1 (GLP-1) receptor agonism, mammalian forkhead transcription factors of the “O” class (FoxOs), and mitochondrial dynamics can provide new insights into the mechanisms that bridge dementia and mood disorders together.
- Future considerations must address the high complexity of these pathways, their dependence upon one another, and the critical modulation of individual components that can affect clinical care.

## Box 2: Current Mechanistic Challenges

- A balance in the activity of autophagy is necessary since under some conditions, such as with high levels of glucose, autophagy dysregulation occurs that can result in cell injury.
- Heightened activity of autophagy can lead to diabetic retinopathy, block cerebral interneuron progenitor cell growth, prevent neuronal cell repair, worsen depression, and impair cognition.
- Pathways that can modulate autophagy, such as the mechanistic target of rapamycin (mTOR), are critical to preserve cellular survival and clinical function with its inverse relationship to autophagy.
- Autophagy and mTOR form a biological connection necessary for effective clinical outcomes.
- Activation of autophagy in experimental models can reduce oxidative stress, increase survival of neurons, limit amyloid (Aβ) deposition in the brain, preserve mitochondrial function, reduce depression, and prevent cognitive impairment.
- Yet, with exaggerated levels of autophagy, tauopathy can progress with cognitive loss, increased neuronal damage can result, depression ensues, and depressive-type phenotypes are promoted.
- Although experimental models support the role of glucagon-like peptide-1 (GLP-1) receptor activation to reduce depression and improve cognition through mTOR activation, GLP-1 agonism does not consistently function through mTOR, such as to reduce retinopathy, but also employs autophagy induction with a corresponding necessary reduction in mTOR activity.
- Careful modulation of cellular mammalian forkhead transcription factor (FoxO) pathways also can be vital to overall cell survival in the nervous system, since FoxOs can be required to promote cellular growth, cellular maintenance, and regulate innate immunity.
